# A Rare Case of an Undiagnosed Middle Ear Tumor Due to Late Referral

**DOI:** 10.7759/cureus.12584

**Published:** 2021-01-08

**Authors:** Jia Ji Ng, Hui Yan Ong, Zara Nasseri, Mohd Imree Azmi, Asma Abdullah

**Affiliations:** 1 Otolaryngology - Head and Neck Surgery, University Kebangsaan Malaysia Medical Center, Kuala Lumpur, MYS; 2 Otolaryngology - Head and Neck Surgery, University Malaysia Medical Centre, Kuala Lumpur, MYS; 3 Otolaryngology - Head and Neck Surgery, Universiti Kebangsaan Malaysia Medical Centre, Kuala Lumpur, MYS; 4 Radiology, Universiti Kebangsaan Malaysia Medical Centre, Kuala Lumpur, MYS

**Keywords:** facial nerve paralysis, facial nerve schwanoma, middle ear tumors, paraganglioma

## Abstract

Facial nerve tumors constitute about 5% of all facial nerve paralysis. As it is relatively uncommon, it could be misdiagnosed. We encountered an 18-year-old girl who had right facial weakness since the age of four, referred to otorhinolaryngology clinic for further evaluation only when her hearing deteriorated and the facial weakness worsened. Further investigation revealed facial nerve schwannoma. Facial nerve paralysis in the pediatric age group is uncommon and should be examined in detail to rule out other possible etiologies besides Bell’s palsy.

## Introduction

Middle ear tumors are rare neoplasms with variable disease presentations and manifestations. The incidence is reported to be only one in 1.3 million people per year for a glomus tumor, which is the commonest type of middle ear tumor [1]. Due to its rarity, especially in the pediatric age group, it is often misdiagnosed, especially in the primary care setting. The delay in diagnosis may eventually lead to more debilitating complications. 

## Case presentation

An 18-year-old girl with right facial asymmetry since the age of four presented with complaints of worsening right facial asymmetry for three months, ipsilateral reduced hearing as well as ipsilateral non-pulsatile tinnitus for the past year. There was no associated vertigo, headache, nor other neurological symptoms.

**Figure 1:**
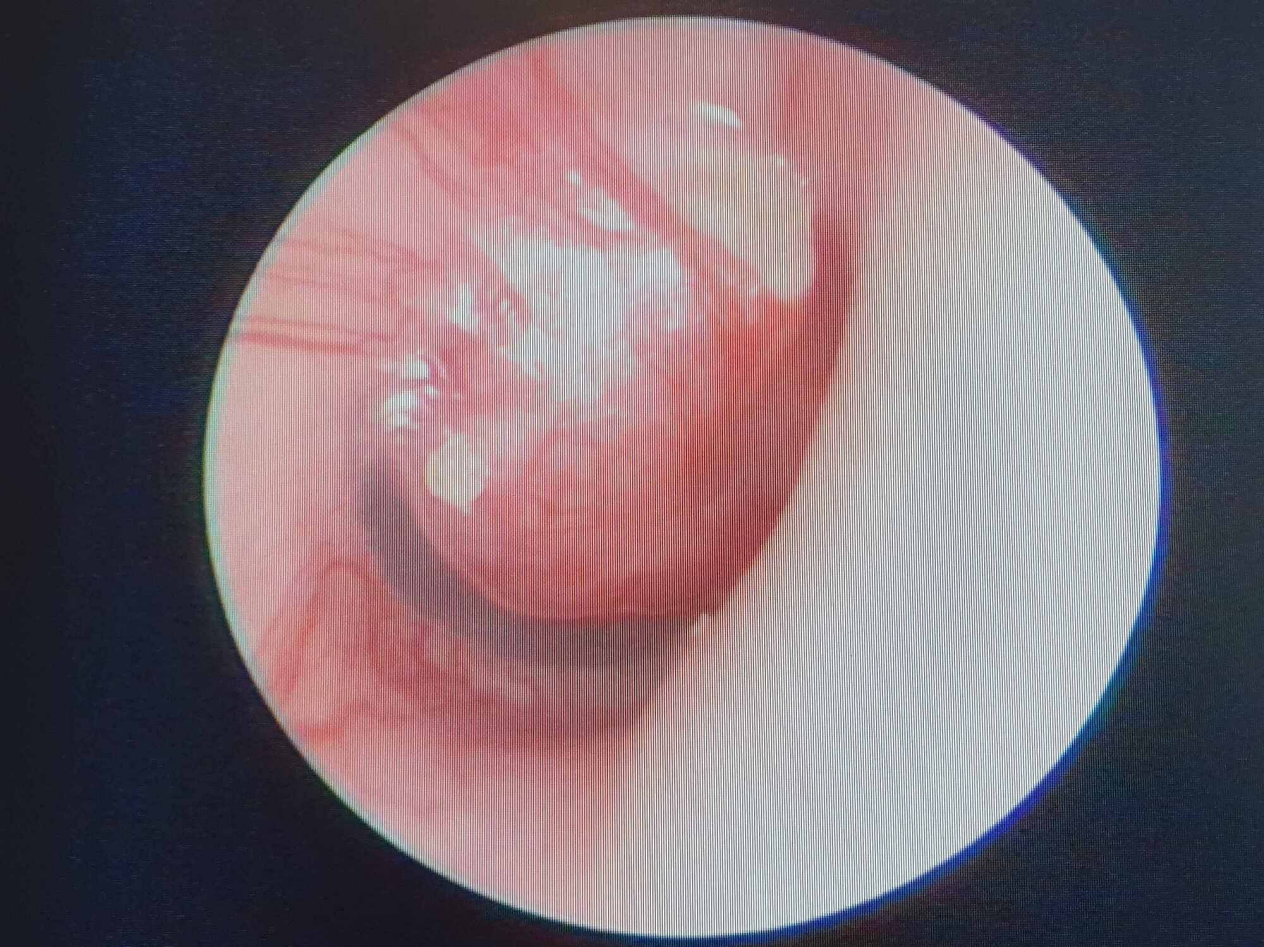
Otoscopic findings of the right ear Pinkish mass is seen medial to an intact tympanic membrane.

Her vital signs were normal, with stable blood pressure and heart rate. She was noted to have right House-Brackmann (HB) grade V lower motor neuron facial nerve palsy. Bell’s phenomenon was observed. Other examinations of cranial nerves were intact. Otoscopic examination of the right ear showed a non-pulsatile reddish mass medial to an intact tympanic membrane (Figure 1).

The mass did not blanch on pneumatic otoscopy; Brown’s sign was negative. There was no cervical lymphadenopathy. Pure tone audiometry of the right ear revealed moderate to severe mixed hearing loss with tympanogram Type B, while the left showed mild sensorineural hearing loss with tympanogram Type A.

**Figure 2:**
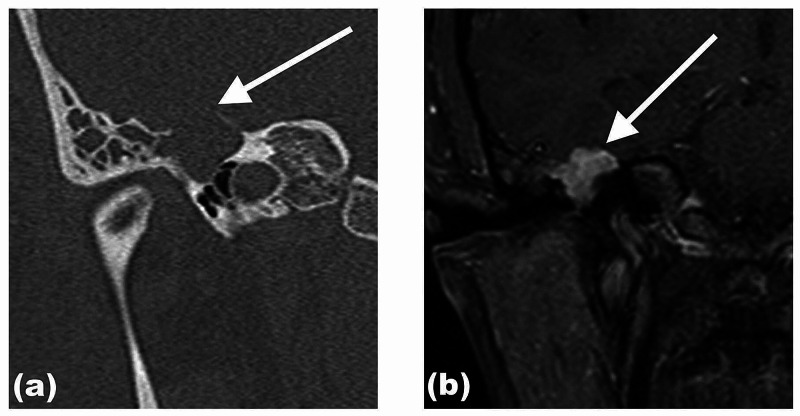
Coronal reformatted view of HRCT of the right temporal bone (a) and contrast-enhanced T1-weighted image (b) Coronal reformatted view of HRCT of the right temporal bone (a) and contrast-enhanced T1-weighted image (b). The facial nerve schwannoma is causing a widening of the epitympanic space (a, white arrow) and extending into the mastoid air cells. On MRI, there is enhancement seen of the facial nerve schwannoma and extension to the middle cranial fossa as extradural mass (b, white arrow). HRCT - high-resolution computed tomography

**Figure 3:**
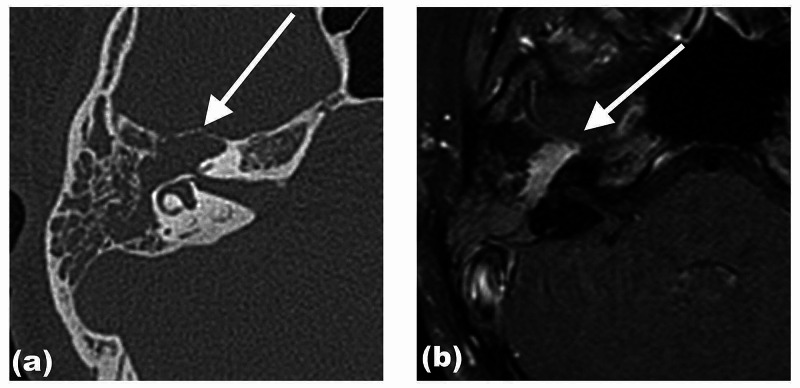
Axial view of HRCT of the right temporal bone (a) and contrast-enhanced T1-weighted (b) There is an expansion of the genu and tympanic segments of the right facial canal associated with bone dehiscence (a, white arrow). Note the enhancement of the facial nerve schwannoma, involving the genu and tympanic segments sparing the labyrinthine segment (b, white arrow). HRCT - high-resolution computed tomography

**Figure 4:**
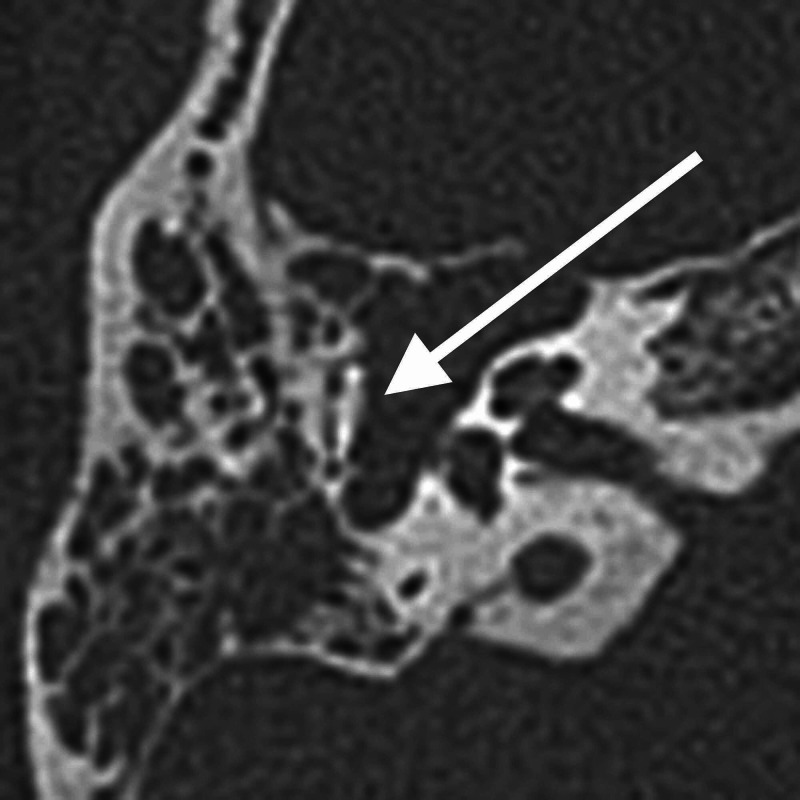
Axial view of HRCT of the right temporal bone The lesion is displacing the malleus and incus superolaterally. Note the erosion at the incudomalleolar joint (white arrow). HRCT - high-resolution computed tomography

Her high-resolution computed tomography (HRCT) of the temporal bone was done and showed a right middle ear soft tissue lesion occupying the right mastoid region, with the erosion of the epitympanum and the incudomalleolar complex. There was also smooth widening and dehiscence of the facial nerve bony canal (Figures 2-4). Incidentally, an old transverse fracture of the right mastoid air cells, involving the posterior wall of the jugular foramen, was seen. Magnetic resonance imaging was done subsequently, and an enhancing lobulated lesion in the right middle ear seen in T1 weighted sequence post gadolinium was suggestive of facial nerve schwannoma.

A transmastoid facial nerve exploration and the facial nerve tumor was removed macroscopically. Intra-operatively, the mass was seen arising from the tympanic segment of the facial nerve, extending proximally to the first genu and distally to the proximal part of the vertical segment of the facial nerve. Histopathological analysis confirmed the diagnosis of facial nerve schwannoma. Postoperatively, her right facial nerve was HB grade VI, despite facial physiotherapy. The patient then underwent right facial nerve repair with an ansa hypoglossi nerve transfer to improve her facial nerve function. She eventually was referred to the ophthalmology team for lateral canthopexy to improve her eye closure. A repeated hearing assessment three months postoperatively showed a similar level of hearing loss, with no further deterioration.

## Discussion

Facial nerve paralysis, especially when occurring in children, is alarming and requires medical attention due to various possible etiologies, as well as its functional and aesthetic implications. Though Bell’s palsy is the commonest cause of facial nerve paralysis in children, if the onset is gradual or prolonged, it may be suggestive of a neoplastic etiology. Rarely, it could be due to tumors such as schwannomas and haemangiomas of the facial nerve or bone tumors such as rhabdomyosarcoma and histiocytosis [2]. In this case, our patient presented with facial nerve asymmetry since the age of four years old but was not investigated until she developed progressive ipsilateral reduced hearing.

Facial nerve tumors contribute to approximately 5% of all facial nerve paralysis. Facial nerve schwannoma is the most common facial nerve tumor [3]. It may arise anywhere along the course of the facial nerve, from its origin in the cerebellopontine angle to its extracranial branches. The clinical presentation mainly depends on the location of the tumor along the facial nerve. Typically, facial nerve schwannoma presents with peripheral nerve neuropathy, such as weakness, focal twitch, or full hemifacial spasm. Hearing loss, middle ear mass, and vestibular findings are also commonly reported [4].

Hearing loss associated with facial nerve schwannomas could be either sensorineural, due to compression of the vestibulocochlear nerve, or if the cisternal or intracanalicular segments are affected; conductive if the intratympanic segment of the facial nerve is affected [5]. In this case, the hearing loss is predominantly sensorineural as there was vestibulocochlear nerve infiltration, suggested by the enhancement of the nerve in the imaging. Although there was minimal erosion of the incudomalleolar joint, the degree of vestibulocochlear nerve involvement was more prominent.

Imaging is essential to delineate the location of the lesion. Topographic localization would help in determining the choice of imaging. MRI is superior to HRCT temporal bone to localize intracranial involvement or if the mastoid portion is involved, whereas HRCT temporal visualizes the tympanic segment of the facial nerve better [5]. Facial nerve schwannoma is mostly described as a well circumscribed, smooth lesion, which appears iso- to hypointense in T1-weighted images and hyperintense on T2-weighted images. Smooth widening of the facial nerve canal and pressure erosion of the ossicles and the bony labyrinth are among the characteristics of facial nerve schwannomas. It may be difficult to differentiate from vestibular schwannoma if the lesion involves the cerebellopontine cistern segment or the internal auditory canal segment [6].

Currently, conservative or facial nerve preservation surgery is preferable compared to non-facial nerve preservation surgery. The main aim is to preserve facial function at the most favorable level for the longest period. Eshraghi et al. concluded that wait and watch management results in the best facial nerves outcomes [7]. In patients with favorable facial nerve function (HB<III) and stable tumor size, observation with serial imaging is preferred until the facial nerve function deteriorates. When there is worsening of the facial nerve function (HB grades III and IV), and the tumor is slow-growing, stereotactic radiosurgery and surgical decompression should be considered. Whereas in patients with good facial nerve function but with growing tumors, observational management may be appropriate, especially for older patients, whilst stereotactic radiosurgery may be offered to younger patients. Stereotactic radiosurgery is gaining popularity as it has shown good short-term tumor control rates; while preserving remaining facial nerve function without the need for surgery. Surgical resection and reanimation are generally reserved for patients with poor facial function (HB>IV) and large tumors compressing the brainstem [8].

Perez et al. and Shirazi et al. reported on their experience in the surgical management of facial nerve schwannoma [9, 10]. Most who underwent surgical resection had facial nerve HB grade more than three, and nerve grafting used were with sural or greater auricular nerve. The majority of the patients’ facial nerve function and hearing level remained unchanged postoperatively.

For the reported patient, surgical resection was chosen as her facial nerve function deteriorated quickly over a period of three months, which resulted in facial nerve paralysis of HB V. The decision was taken after a multidisciplinary team discussion; also involving the patient and her family, to improve her facial nerve function. As the facial nerve function was grade V prior to the operation, we regretted that it did not improve post removal of the tumor nor with the follow-up surgery. The patient and her family were also well informed regarding the risks of the operation. 

## Conclusions

Facial nerve paralysis in the pediatric age group is uncommon and should be examined in detail to rule out any underlying pathology, more so if there is associated hearing loss. Investigation such as contrasted HRCT of the temporal bone or MRI is crucial to help in early diagnosis. Complications of the disease, such as facial nerve or hearing impairment, can be prevented and managed if the diagnosis is established early. Treatment options should be individually discussed with patients and their family. As facial nerve function directly affects a patient’s quality of life, the patient should be involved in the decision-making process. Other important factors, including the patient’s age, facial nerve function, preoperative hearing status, and tumor size, should also be explored before deciding the best management option.
